# Development and validation of a clinical score for prognosis stratification in patients requiring antiretroviral therapy in sub-Saharan Africa: a prospective open cohort study

**DOI:** 10.4314/pamj.v10i0.72210

**Published:** 2011-09-16

**Authors:** Rivero Gerardo, Pérez Dayana

**Affiliations:** 1 Cuban Medical Brigade Doctor, Opportunistic Infectious Clinic, Parirenyatwa Groups of Hospitals, Harare, Zimbabwe; 2 Infectious Diseases Service, Gustavo Aldereguia Lima Hospital, Cienfuegos, C.P. 55100, Cuba; 3 Hematology Service, Juan Manuel Márquez Pediatric Hospital, 31th Ave. Marianao, La Habana, Cuba

**Keywords:** Africa, antiretroviral therapy, highly active, follow-up studies, HIV, mortality, clinical score, prognosis stratification, lost to follow up, Zimbabwe

## Abstract

**Background:**

Mortality rates among patients initiating antiretroviral therapy (ART) in sub-Saharan Africa continue high. Also HIV treatment services from the region are affronting the challenges of been attending more patients than never. In this scenario, there are no integrated scoring systems capable of an adequate risk identification/ prognostic stratification among patients requiring ART; in order of optimize actual programmes outcomes. Several independent risk factors at baseline are associated with a poor prognosis after ART initiation. These include: male sex, low body mass index, anemia, low CD4 count and stage-4 WHO disease. The aim of this research was evaluate prospectively a new scoring system composed by these factors.

**Methods:**

An open cohort study was conducted in 1769 patients from May 2008 to December 2010 at two HIV clinics of Zimbabwe. A new clinical model (MASIB score) was applied at ART initiation and patients were followed for 4 months. After that, validation characteristics of the score were examined.

**Results:**

Patients selected in this cohort exhibited similar baseline characteristics that the patients selected in previous cohorts from the region. Overall performance for mortality prediction of MASIB score was accurate, as reflected by the Brier score test result 0.084 (95%CI: 0.080–0.088). Calibration was adequate taking in consideration a p>0.05 in the Hosmer Lemeshow test and discrimination was also good (Area Under Curve: 0.915, 95%CI: 0,901– 0,928).

**Conclusion:**

The new model developed exhibited adequate validation characteristics supporting the clinical use. Further evaluations of this model in others scenarios from the sub-Saharan region are needed.

## Background

Thirty years after the beginning of HIV/AIDS pandemic, more people are receiving antiretroviral therapy in the world than at any previous time in the past [1]. In sub-Saharan Africa, nearly 37% of people eligible for treatment were able to access life-saving medicines in 2009; seven years ago it was only 2% [1,2]. The effect of this is now evident: 20% fewer people died of AIDS-related causes in 2009 than in 2004, when antiretroviral therapy began to be dramatically expanded in the region [1]

Despite of progresses, early mortality rates in sub-Saharan Africa are very high; between 8 and 26% of patients die in the first year of antiretroviral treatment (ART), with most deaths occurring in the first 4 months [3]. These high rates may even be underestimates since a substantial proportion of patients from sub-Saharan cohorts initially classified as “lost to follow up” (LTFU) have actually died [4,5]. The reasons behind this problem are many and adequately discussed by others [3,4]. Some of them, however, may reflect weaknesses in early HIV diagnosis and longitudinal HIV care pre-ART since early mortality rates are strongly associated with the degree of immunodeficiency in patients at the time they enroll into ART programmes [3]. Combinations of delays in patient referral, waiting lists for ART initiation and time taken to prepare patients to start life-long treatment are also contributing to the reported mortality rates [3].

On the other hand, the new World Health Organization (WHO) guidelines recommends now to start treatment at an earlier stage (CD4 count of 350 or lower) [6]. Although desirable and based in the best evidence available, it will represent significant increments in the estimated number of people needing antiretroviral therapy in terms of millions of patients [7]. Consequently, HIV treatment services from the sub-Saharan region are affronting the challenges of been receiving, treating and following more patients than never; with greater differences among clinical staging, immunological profile and prognosis. How to balance this in order of optimize actual programmes outcomes requires urgent research attention.

Up to now, there are no integrated scoring systems capable of stratifying HIV/AIDS patients according to its prognosis at the time of health system entry. Prognostic models have long been accepted as useful decision aids for clinicians, in particular by identifying patients who are at high or low risk of death [8]. Adequate risk identification/stratification of the current heterogeneous group of patients requiring ART from the most sub-Saharan Africa antiretroviral programmes brings the opportunity of improving pre-ART care, since time to ART initiation could be favorably managed among selected high risk patients. Once in ART, it might help by focus clinicians and policy makers on a more homogenous group of high risk patients, giving a closer/differentiate therapy, monitoring and follow up to them. A better optimization of human and monetary resources could be therefore possible throughout the process as well.

Whereas the key long-term determinant of mortality is the response to ART, mortality in the first 4 months after therapy initiation is associated with several independent risk factors at baseline [3]. According to Lawn et al. [3, 4], the most consistent clinical factors at baseline in cohorts studies from the sub-Saharan region published from 2002 to 2008 were: male sex, low body mass index, anemia, low CD4 count and stage 4 WHO disease [9–26]. Our hypothesis was that the above mentioned risks factors, could be integrated in a new clinical scoring system designed for the purpose of prognosis stratification among patients requiring ART, at the time of entry into the differents health systems from the region.

## Methods

### Patient and setting

An open cohort study was conducted from May 2008 to December 2010 at the Opportunistic Infectious Clinics (OICs) from Parirenyatwa Groups of Hospitals in Harare City and Chinhoyi Provincial Hospital in Mashonaland West Province, both in Zimbabwe. A total of 2054 patients were initially included in the overall cohort but the model was finally validated using information from 1769 patients that initiated ART and were not excluded during the observation period of 4 months.

The source of the information was the individual medical files of each OIC patient selected according to our inclusion/exclusion criteria. The fulfillment of these clinical records was carried out by the trained Zimbabwean staff of the OIC. Those records were maintained on all patients screened on entry to the ART programme. Information was transferred to a database designed for the purpose of this research, with previous authorization of local authorities (see ethics).

### Inclusion or exclusion criteria for patient selection

1) Age ≥ 18 years; 2) No pregnancy throughout the duration of the study period; 3) CD4 count available at ART initiation; 4) ART started according to the medical criteria contemplated in the 2007 Guidelines for Antiretroviral Therapy in Zimbabwe [27]: WHO [28] clinical stage 3 plus CD4 count less than 350 cells/µl or WHO clinical stage 4 irrespective of the CD4 count or CD4 count<200 cells/µl irrespective of the WHO staging; 5) ART combinations and visits schedule according to the 2007 Guidelines for Antiretroviral Therapy in Zimbabwe [27]; 6) Inclusion only for ART-naïve patients; 7) MASIB score punctuation available at ART initiation (see model development); 8) Exclusion for patients lost to follow up (patients were define arbitrarily as lost to follow up if last contact was>1 months before the end of the study); 9) Exclusion for patients in which the direct causes of death were not related to HIV/AIDS or were not accessible for the researchers.

### ART definition

Antiretroviral therapy was defined as a regimen of at least 3 drugs from the following groups: nucleoside reverse transcriptase inhibitors (NRTIs), non-nucleoside reverse transcriptase inhibitors (NNRTIs), nucleotide reverse transcriptase inhibitors (NtRTIs) and proteases inhibitors (PIs).

### Clinical End Point

Vital status was assessed through 30 days and every 1 month until follow up completion. The primary end point of the cohort was death within 4 months of follow up. Mortality data were obtained through telephone follow-up, OIC records or outpatient visitation.

### Predictors and Model Development

We examined a set of 5 variables which constituted independent risk factors for early mortality after ART initiation according to the results of 18 cohorts studies published from 2002 to 2008 in the sub-Saharan region [3,9–26]. These included: male sex (**M**), anemia (**A**), stage 4 WHO disease (**S**), low CD4 count (**I**), and low body mass index (**B**); conforming the **MASIB** score.

We assigned points for each variable based on the median adjusted hazard ratios (AHR) from the multivariable model of the cohorts studies above mentioned. The median AHR were rounded to the nearest number to determine the points. The MASIB risk score was developed by summing the points for each risk factor present. We applied the score in every selected patient at ART initiation.

### Data management and statistical analysis

Statistical analyses were carried out using SPSS software for Windows, version 15.0 (SPSS Inc., Chicago, IL, USA) and MedCalc version 11.4.0. Categorical values were expressed in absolute and relative frequencies, and were analyzed using x^2^ test. Continuous variables were presented as median (25–75% interquartile range) and compared using non parametric test of Kruskal Wallis of variance analysis. Student t was also used. Survival analysis was performed using Kaplan Meier curves. For establish curves differences, the Log-Rank (Mantel-Cox) test was used. The signification level was adjusted at α=0.05. Validation of the prognostic score was performed using standard tests to measure discrimination, calibration and overall performance:

1) Ability to identify those at high risk (discrimination). This was assessed from the area under the receiver operating characteristic curve (AUC; c statistic), with discrimination considered perfect if AUC=1, good if AUC>0.8, moderate if 0.6–0.8, poor if <0.6, and no better than chance if AUC=0.5; 2) Ability to quantify risk (calibration or goodness-of-fit — how close the predicted probabilities are to the observed outcome).This was assessed by the Hosmer–Lemeshow C-test, which divides the cohort into deciles of predicted risk and compares these with actual outcomes. A P value of less than 0.05 in the Hosmer Lemeshow Chi-squared C statistical test indicates a signi?cant departure from the null hypothesis of ‘no difference between the observed and predicted values’, suggesting poor calibration; 3) Overall performance (how well the model predicts the likelihood of an outcome in an individual patient). This was assessed by the Brier score (mean squared error). Brier score (BS) is calculated as ? (yi– pi)2/n, where y denotes the observed outcome and p denotes the predicted probability for subject i in the data set of n subjects. Brier score (BS) ranges from 0 to 0.25, with a Brier score of zero signi?es a perfect prediction model and a Brier score of 0.25 signi?es a useless prediction model [29,30].

For selection of the point in the scale in which patients are at higher risk for death after ART initiation, we also examined the sensitivity, specificity, positive and negative predictive values, and positive and negative likelihood ratios in selected cut-off points of the MASIB score.

### Ethics

This study was conducted according to the principles of the Declaration of Helsinki. General measures were taken in order to ensure patient confidentiality. Authors obtained work authorization in Zimbabwe by the Ministry of Health and Child Welfare before the commencement of this research. Authors obtained permissions letters from the Medical Superintendent of Chinhoyi hospital and from the Clinical Director of Parirenyatwa Groups of Hospitals in order of allowing the access to the OIC files by the researchers. There were no interferences in the standard management and patients follow up.

## Results

### Model development

Table 1 summarized evidences from 18 selected cohort studies (36204 patients) from sub-Saharan Africa, reporting risk factors for early mortality after ART initiation [3,5–22]. A total of 4 cohorts supported anemia and male sex as independent risk factors with a median of adjusted hazard ratio (AHR) from the multivariate analysis of 2.8 and 1.6 respectively. A number of 8 cohorts (31179 patients involved) supported a CD4<50 cells/µl as an independent risk factor with a median AHR of 2.7. Eleven cohort studies (35 408 patients) reported evidence for stage 4 WHO disease (median AHR: 2.8) and seven cohort studies (33174 patients) reported a low BMI (<18.5 kg/m^2^) as independent risk factor (median AHR: 2.4). Thus, median AHRs were rounded conforming MASIB score: 1.5 points for male sex, 2.5 points for low BMI and 3 points for the rest of the 3 variables (see Table 1).

**Table 1:** Independent predictors for early mortality after ART initiation in Sub-Saharan cohorts from 2002 to 2008 and weights (points) for the MASIB score

Set of baseline clinical independent risk factors [3, 4]	Supporting evidence in Sub-Saharan cohorts*	Patients involved	Median (IQR) of AHR for death	Points allocated (MASIB-score)
Male sex	6, 10, 11, 22	15 280	1.6 (1.5–1.9)	**1.5**
CD4 count <50 cells/µl	5, 6, 7, 9, 11, 12, 13, 17	31 179	2.7 (2.2–3.1)	**3**
WHO stage 4 disease	5, 6, 7, 9, 10, 11, 13, 15, 16, 17, 22	35 408	2.8 (2.1–3.4)	**3**
Low BMI<18.5 kg/m^2^	5, 6, 11, 15, 17, 18, 22	33 174	2.4 (1.9–2.9)	**2.5**
Anaemia <8 g/l	5, 6, 15, 18	27 521	2.8 (1.5–7.6)	**3**

IQR, interquartile range; AHR, adjusted hazard ratio;

* numbers represents references.

### Baseline patient characteristics

Between May 2008 and December 2010, 2054 patients were initially included in the overall cohort. Of these, the following were excluded from the study analysis: LTFU patients (n=223), deaths causes not related to HIV/AIDS or not accessible for the researchers (n= 54), new pregnancy (n= 8). The remaining 1769 subjects finished the follow up and were included in this analysis. Of these, 413 (23.3%) resulted death at the study closing date. Demographic data for these patients are summarized in Table 2.

**Table 2:** Baseline characteristics and starting ART regimens in patients included in study

Baseline characteristics	Total cohort	Survivors	Non-survivors
No. of male (%)	666 (37.6)	425 (31.3)	241 (58.4)
Age, median (IQR), in years	38 (31–43)	37.61 (31.4–42.8)	37.13 (31.1–42.6)
Body mass index, median (IQR), in kg/m^2^	20 (17–23)	20.9 (17.3–23.1)	18.5 (16.1–21.6)
			
**CD4 cell count**			
Median (IQR), in cells/µl	117 (50–204)	162.1 (106–256)	55.9 (25.2–113.3)
Count<50 cells/µl, in n (%)	439 (24.8)	183 (13.5)	256 (62.0)
			
**WHO clinical stage, number of patients (%)**			
1 and 2	236 (13.3)	234 (13.2)	2 (0.1)
3	1105 (62.5)	982 (55.5)	123 (7.0)
4	428 (24.2)	140 (7.9)	288 (16.2)
			
Haemoglobin levels, median (IQR), in g/l	11.2 (10.7–11.8)	11.3 (9.9–12.6)	8.8 (6.3–11.5)
Patients on co-trimoxazole prophylaxis: n (%)	1711 (96.7)	1323 (97.6)	388 (93.9)
			
**ART regimen**			
NNRTI-based n (%)	1760 (97.8)	1356 (100)	404 (97.8)
NVP based: n (%)	1288 (73.2)	1168 (86.1)	120 (29.7)
EFV based: n (%)	472 (26.8)	188 (13.9)	284 (70.3)
PI-based	9 (2.2)	-	9 (2.2)
Median follow-up time (IQR), in weeks	16 (15.7–16.0)	16.0 (16.0–16.0)	5.71 (1.23–8.34)

ART, antiretroviral therapy; IQR, interquartile range; NNRTI, non-nucleoside reverse transcriptase inhibitors; NVP: nevirapine; EFV: efavirenz; PI, protease inhibitors.

The median age for the cohort was 38 years (IQR: 31–43) with no differences between survivors and no survivors. Median CD4 count for the cohort was 117 cells/µl, haemoglobin levels 11.2 g/l and BMI was 20 kg/m^2^; with significant differences among medians between survivors and no survivors (Table 2). Six hundred sixty-six patients (37.6%) of the cohort were male. A total of 428 patients were classified as stage 4 WHO disease, and of those 288 (67.3%) resulted death at the end of the observation period. Most of the patients were on cotrimoxazole prophylaxis and in the first line non-nucleoside reverse transcriptase inhibitors regimen (see Table 2).

### Model performance

**Figure 1:**
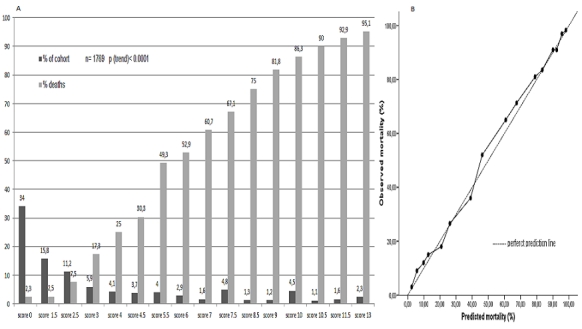
MASIB risk score for predicting 4 months mortality. Panel A: Bar chart representing absolute frecuencies (in percentages) by cut off points. Panel B: Calibration curve of MASIB score (x2= 4.62; p= 0.71)

The MASIB risk score (Figure 1) showed a strong association with mortality at 4 months, with a graded increase in mortality between those with a risk score punctuations of 0 and those with a score punctuations of 13 (P(trend)<0.0001). More than 85% of the people resulted death when initial MASIB score punctuation was 10 points or more. On the other hand, mortality was an infrequent event in punctuation <1.5 points (Figure 1).

**Figure 2:**
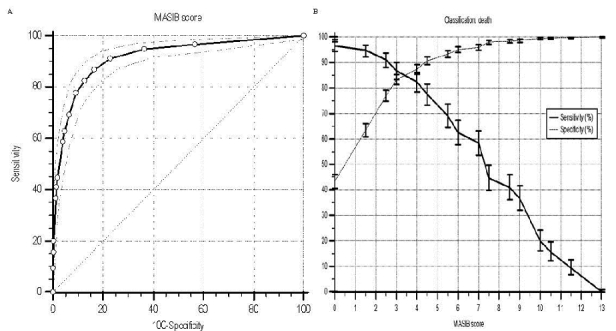
Receiver operating characteristic (ROC) curves analysis. Panel A: ROC curve performance (95% CI). Panel B: Sensitivity and specificity (95% CI) plotted against the criterion values

In relation to discrimination properties, Figure 2 represents ROC curves analysis. Figure 2 and Table 3 shows the graphical representation and numeric value of the area under the curve (AUC) for MASIB score, respectively. The AUC of the MASIB model was 0.915 (95% CI: 0,901– 0,928). Also AUC comparisons (graphs not showed) were carried out using the test of Hanley and McNeil. In all cases, the AUC from the full model was significant higher compared with AUCs generated by partials models that included combinations of 3 variables (WHO stage 4 disease and CD4count <50 cells/µl plus one of the others 3 variables). Calibration as tested by C-statistics showed no significant differences between predicted and observed mortality (Figure 1, Table 3). The overall performance (Table 3) calculated by BS was 0.084 (95% CI: 0.080–0.088).

**Table 3:** Discrimination and model performance statistics for MASIB score in estimating HIV/AIDS related deaths after 4 months of ART

		Performance statistics						
				**Discrimination**		**Calibration**		**Overall performance**

**Model**	**Number of Patients**	**Outcome**	**No. of events**	AUROC		H-L test		BS(95% CI)

			AUC (95%CI)	p	C-test	p		
					
**MASIBscore**	1769	Deaths related HIV/AIDS	413	0.915(0,901–0,928)	0.000	4.62	0.71	0.084(0.080-0.088)

		**Selected cut off points MASIB score**						

**Operating characteristics**		**≥0**	**>1,5**	**>2,5**	>3*	**>4**	**>4,5**	**>5,5**

Sensitivity (95%CI)		100(99,1–100)	94.9(92,3–96,8)	91,3(88,1–93,8)	**86,9(83,3–90,0)**	82,6(78,6–86,1)	77,7(73,4–81,6)	69,3(64,6-73,7)
Specificity (95%CI)		0(0,0–0,3)	63,5(60,9–66,1)	77,1(74,7–79,3)	**83,4(81,3–85,3)**	87,4(85,5–89,1)	90,8 (89,1–92,3)	93,4 (92,0–94,7)
+LR (95%CI)		1(–)	2.6(2,5–2,7)	3.98(3,8–4,1)	**5.24(5,0–5,5)**	6.55(6,2–6,9)	8,43(8,0–8,9)	10,55(9,9–11,3)
−LR (95%CI)		−(–)	0,08(0,05–0,1)	0,11(0,08–0,2)	**0,16(0,1–0,2)**	0,2(0,2–0,3)	0,25(0,2–0,3)	0,33(0,3–0,4)
+PV (95%CI)		23,3(21,4–25,4)	44,2(40,9–47,5)	54,8(51,0–58,6)	**61,5(57,4–65,4)**	66,6(62,3–70,7)	72(67,6–76,1)	76,3(71,6–80,5)
−PV (95%CI)		−(–)	97,6(96,4–98,5)	96,7(95,4–97,7)	**95,4(94,1–96,6)**	94,3(92,8–95,5)	93(91,5–94,4)	90,9(89,3–92,3)

AUROC, area under receiver operating curve; H-L, Hosmer–Lemeshow; BS, Brier Score; +LR, Positive likelihood ratio; −LR, Negative likelihood ratio; +PV, Positive predictive value; -PV, Negative predictive value;

* Cut off point recommended by MedCalc software.

For the selection of the cut-off in the model, ROC curves (Figure 2) and operating characteristics analyses (Table 3), were performed. MedCalc software recommended a cut-off ≥3, showing at this point a sensitivity of the model of 86,9 (95%CI: 83,3–90,0), an specificity of 83,4 (95%CI: 81,3–85,3), a positive likelihood ratio of 5,24 (95%CI: 5,0–5,5), a negative likelihood ratio of 0,16 (95%CI: 0,1–0,2) and a positive and negative predictive values of 61,5 (95%CI: 57,4–65,4) and 95,4 (95%CI: 94,1–96,6), respectively.

### Survival analysis

**Figure 3:**
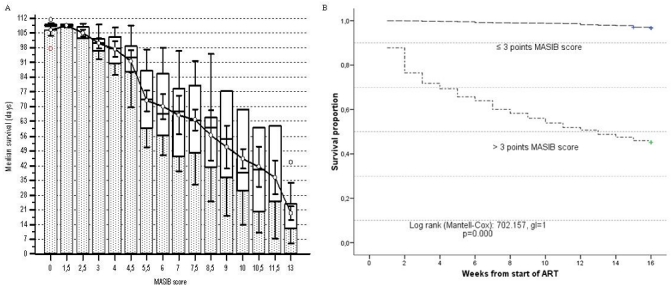
Actuarial survival analisys acording to MASIB score. Panel A: Boxplots and whiskers graph displaying median survival diferences according score puntuation (Kruskal Wallis test= 257,2641, P<0, 0001). Panel B: Survival differences according Kaplan Meier curves for cut off at 3 points

Figure 3 represents the impact on survival of patient's stratification according to initial MASIB score punctuations. Figure 3a shows the graphic representation of the Kruskal Wallis test comparing median survivals (days) by punctuations groups. Medians survivals (days) were significant lower with graded increase in initial punctuations (Kruskal Wallis test= 257, 2641, p< 0, 0001). On the other hand (Figure 3b), survival was significant lower in patients with initial punctuations of ≥3 points (Log-rank= 702.157, p= 0.000).

## Discussion

### Statement of principal findings

Despite the seriousness of this public health problem in the most countries from sub-Saharan Africa, very little is reported in relation to systematize the well documented risk factors in order of stratifying patients at ART initiation. The present study sheds some light on this problem.

The main findings in the validation of MASIB score can be summarized as follows. First, the overall performance for mortality prediction was accurate, as reflected by the Brier score test nearly to 0. Second, calibration was adequate taking in consideration a p>0.05 in the Hosmer Lemeshow test and third, discrimination was good (AUC>0.8). Due to the fact of good performances characteristics of the model, survival was adequately described, with significant reduction in the survival with graded increase of initial score punctuations. It is remarkable the added clinical benefit from doing a composite score of 5 variables since discrimination proprieties of the full model is significant better compared with partials models of fewer variables. This suggests that clinicians should not continue using only clinical and immunologic stage to predict patient prognosis.

Patients LTFU were excluded in this initial validation study of MASIB score. We were not able to conduct an intensive active follow up among LTFU patients in order of establishing the vital status, time to death and deaths related causes because of economics and ethical reasons. However, taking in consideration that losses to follow-up occur in a high proportion of patients initiating ART and it is now well appreciated that these losses lead to substantial underestimation of mortality [31], future evaluations of the MASIB model may interestingly include the analysis of this particular group of patients.

Our results suggest that patients with initial punctuations of 3 or more points in the score should be managed differently compared with the standard care of those with fewer points. It should include urgent referral to ART clinics, avoiding waiting list for ART initiation and perhaps a shorter or differentiate counseling process. Patients with very high punctuations on the score may even be hospitalized. However intensifying case management for such a large proportion of ART initiations is no small task. In our study, patients with initial punctuations of 3 or more points represented almost 40% of overall cohort. This important finding describe how late a large proportion of patients are initiating ART, reinforcing the fact that strategies to reduce mortality must therefore focus not only on delivery of care within ART programmes but more fundamentally they must promote early HIV diagnosis and improved pre-ART HIV care [3,4].

Although validated in a prospective cohort of patients initiating ART, we recommend the application of MASIB score not only at ART initiation but also at the first contact of patients with HIV care, since the risk factors influencing on mortality seems to be the same. It could also help in maximizing the benefits of this prognostic model.

### Strengths and weaknesses

Our study has the strength of being a prospective cohort, developed in two different health centres of Zimbabwe. The score was conformed taking in consideration the best available evidence from sub-Saharan Africa cohorts studies. Also, our study population (n) is the fourth biggest compared with the 18 reported cohorts from 2002-2008 [3,9–26]. The median CD4 count for the cohort 117 cells/µl (IQR: 50–204) is in the range reported in the above mentioned studies. It was the first time in which a cohort from sub-Saharan Africa takes in consideration patients from both urban and rural settings of a country at the same time. Moreover, the 5 variables that conforms the model are commonly and routinely measured in every patient that is initiated in most ART programmes.

Weaknesses in our study are those inherent to the use of scores. Several authors have debated the limitations of risk factors (or weighted risk factors such as risk scores) as prognostic tools, especially at the individual level [32,33]. That is why in an attempt to use MASIB score to identify high-risk individuals for intervention, we used several statistical tools in order of select the best cutoff possible, capable of been able to separate the low risk individuals from the high risks with relative accuracy. Further researches could be centered in this topic.

Another weakness in our study is the lack of a previously validated model for prognosis stratification after ART initiation; therefore, we were not able of comparing the "certified tool" (validity criteria).

### Unanswered questions for future research

Further evaluations of this model in differents scenarios from the sub-Saharan countries are now required and desirable since others non-clinical risk factors like the need to pay for treatment, macro-economic factors and limitations in healthcare provision; are also influencing on the overall early mortality [4].

The 5 variables selected for the conformation of the MASIB score applies also as independents risk factors for early mortality in patients starting ART in other resource limited setting from the rest of the world [4]; so, it opens the possibility of application of our prognosis score in other places different to the sub-Saharan Africa.

## Conclusion

Patients selected in this cohort exhibited similar baseline characteristics and mortality rates that the patients selected in previous cohorts from the region. The results of this study confirm the experimental hypothesis that a mortality risk score can be developed on the basis of well documented risk factors in patients initiating ART in sub-Saharan countries. Using 5 risk factors, we were able to develop a risk score model that exhibit accuracy, good calibration and satisfying clinical performance. Further evaluations of this model in others scenarios from the sub-Saharan region are needed.
